# Differential effects of ketoconazole on exposure to temsirolimus following intravenous infusion of temsirolimus

**DOI:** 10.1038/sj.bjc.6604376

**Published:** 2008-05-06

**Authors:** J P Boni, C Leister, J Burns, B Hug

**Affiliations:** 1 Department of Clinical Pharmacology, Wyeth Research, 500 Arcola Road, Collegeville, PA 19426, USA

**Keywords:** temsirolimus, cytochrome *P*450, CYP3A4, ketoconazole, pharmacokinetics

## Abstract

Intravenous (i.v.) temsirolimus, a novel inhibitor of mammalian target of rapamycin, is approved for the treatment of advanced renal cell carcinoma and is being studied in patients with mantle cell lymphoma. Because temsirolimus and its primary metabolite, sirolimus, are metabolised by the cytochrome *P*450 3A4 pathway (CYP3A4), the potential exists for pharmacokinetic (PK) drug interactions with the numerous agents that modulate CYP3A4 isozyme activity. We investigated the effects of ketoconazole, a potent CYP3A4 inhibitor, on the PK profile of i.v. temsirolimus in healthy adults. Coadministration of 400 mg oral ketoconazole with 5 mg i.v. temsirolimus had no significant effect on temsirolimus maximum concentration (*C*_max_) or area under the concentration curve (AUC). However, mean AUC increased 3.1-fold and AUC_sum_ (sum of temsirolimus plus sirolimus AUCs) increased 2.3-fold compared with temsirolimus alone. A single 5-mg dose of temsirolimus with ketoconazole was well tolerated, and there were no unexpected safety results. Therefore, in cancer patients receiving 25 mg i.v. temsirolimus, concomitant treatment with agents that have strong CYP3A4 inhibition potential should be avoided. If a concomitant strong CYP3A4 inhibitor is necessary, a temsirolimus dose reduction to 12.5 mg weekly should be considered.

Temsirolimus, a novel inhibitor of the mammalian target of rapamycin (mTOR), improves overall survival and progression-free survival in patients with advanced renal cell carcinoma (RCC) compared with interferon (Hudes *et al*, 2007). Rash, peripheral oedema, hyperglycaemia, and hypertriglyceridaemia were more common in advanced RCC patients receiving temsirolimus, whereas asthaenia occurred more often in patients receiving interferon. *In vitro*, the activity of temsirolimus is facilitated by its binding to the cytosolic FK506 binding protein-12, which in turn binds to mTOR and inhibits its kinase activity (Skotnicki *et al*, 2001). Mammalian target of rapamycin is a central regulator of various signal-transduction pathways, controlling synthesis of proteins that are needed for cell proliferation, growth, survival, and response to hypoxic stress (Schmelzle and Hall, 2000; Hudson *et al*, 2002; Fingar *et al*, 2004). Inhibition of mTOR by temsirolimus as a novel mechanistic strategy for the treatment of other advanced malignancies, including mantle cell lymphoma, is actively being studied in several clinical trials.

The principal metabolite of intravenous (i.v.) temsirolimus in humans is sirolimus, which also displays mTOR inhibitory activity. Clinically relevant pharmacokinetic (PK) exposure to i.v. temsirolimus is considered to be a composite of both temsirolimus and sirolimus. Following administration of a single 25-mg dose of temsirolimus in patients with RCC, temsirolimus mean maximum concentration (*C*_max_) in whole blood was 595 ng ml^−1^ (coefficient of variation (CV)=17%), and mean area under the curve (AUC) was 1580 ng h ml^−1^ (s.d., CV=17%) (Atkins *et al*, 2004). Sirolimus appears rapidly and exhibits *C*_max_ values 10–20% of temsirolimus *C*_max_ values (Atkins *et al*, 2004). However, owing in part to the longer half-life of sirolimus, the sirolimus/temsirolimus AUC_ratio_ indicates a 2.8-fold higher exposure to sirolimus relative to temsirolimus over the course of treatment. Temsirolimus and sirolimus are both cleared by the liver and are extensively excreted in the faeces (Torisel, 2007). Because only small amounts are excreted in urine, dispositions in RCC patients with nephrectomy or renal impairment are not expected to differ substantially.

Temsirolimus and sirolimus are metabolised by cytochrome *P*450 3A4 (CYP3A4) (Zimmerman, 2004; Cai *et al*, 2007); this enzyme is responsible for the formation of five temsirolimus metabolites (Torisel, 2007). Because CYP3A4 is the most abundant CYP isozyme in humans and the pathway responsible for metabolism of numerous xenobiotics (Shimada *et al*, 1994; Li *et al*, 1995; Dresser *et al*, 2000), the potential for drug interaction exists. Using *in vitro* liver microsome systems, ketoconazole, a potent inhibitor of CYP3A4, decreased the formation of temsirolimus metabolites to 10–20% of control levels (IC_50_<2 *μ*M) (Cai *et al*, 2007). Ketoconazole also inhibited the metabolism of sirolimus (*K*_i_=2 *μ*M). Orally administered sirolimus exhibits a substantial PK drug interaction with ketaconazole, resulting in increased whole blood exposure to sirolimus (Zimmerman, 2004). Oral structural analogues of sirolimus (tacrolimus and everolimus) are also CYP3A4 substrates and exhibit strong drug interactions with ketoconazole (Kovarik *et al*, 2005, 2006; El-Dahshan *et al*, 2006).

This drug interaction study was conducted in healthy adults to assess the PK profile of a single i.v. dose of temsirolimus when coadministered with multiple oral doses of the potent CYP3A4 inhibitor ketoconazole Because of the potential for increased exposure when given with ketoconazole, a 5-mg dose of i.v. temsirolimus was chosen to mitigate safety concerns of using the 25-mg clinical RCC dose in healthy subjects. The lower dose is also a more sensitive indicator of PK drug interaction potential.

## MATERIALS AND METHODS

### Subjects

Men and women aged 18–45 years, inclusive, with a body mass index between 18 and 30 kg m^−2^ and body weight of 50 kg or more were eligible for participation in this trial. Women were required to be of non-childbearing potential due to hysterectomy and/or oophorectomy or in menopause for more than 1 year, with an oestradiol concentration of 20 pg ml^−1^ or less and a follicle-stimulating hormone level of at least 40 mIU ml^−1^. They also were required to have a negative serum pregnancy test within 48 h of the first temsirolimus administration (study day 1). Subjects with significant cardiovascular, hepatic, renal, respiratory, endocrine, immunologic, dermatologic, haematologic, neurologic, or psychiatric disease or a surgical or medical condition that could interfere with the absorption, distribution, metabolism, or excretion of the study medications were not enrolled. Those with positive HIV antibodies, hepatitis B surface antigen, or hepatitis C virus antibodies were also excluded. A normal physical exam, vital signs, 12-lead electrocardiogram (ECG), and clinical laboratory tests were required for inclusion in the study. Subjects with an acute illness within 7 days of study day 1 were excluded. A history of smoking fewer than 10 cigarettes per day and the ability to refrain from smoking during the in-patient portion of the trial were required for participation in the study. Subjects were excluded if they had a history of drug or alcohol abuse (daily consumption of more than 24 ounces of beer, 3 ounces of 80-proof alcohol, or 12 ounces of wine) within 1 year of study day 1, and a negative urine drug screen was required. Those with hypersensitivity to temsirolimus or sirolimus, ketoconazole, or diphenhydramine were also excluded. Use of investigational or prescription drugs within 30 days of study day 1 or use of caffeine-containing products, grapefruit, grapefruit-containing products, or alcoholic beverages within 48 h of study day 1 was not allowed. Subjects could not take over-the-counter medications or herbal supplements within 14 days of study day 1. All participants gave written informed consent before the trial.

### Study design

In this open-label, non-randomised, single-centre, two-period, sequential study, subjects received temsirolimus (Torisel™; Wyeth Pharmaceuticals, Philadelphia, PA, USA) alone, followed by temsirolimus in combination with ketoconazole after a 14-day washout period. On study day 1 of the first period, subjects received 25 mg i.v. diphenhydramine, followed 30 min later by 5 mg i.v. temsirolimus over 30 min. Subjects remained in the clinical unit until the 72-h blood collection (study day 4) and returned for subsequent blood draws at designated time points over the next 4 days (study days 5–8). Study days 9 through 13 were part of the washout period. During the second period, subjects returned to the clinical unit on study day 14. On study day 15, they received 400 mg oral ketoconazole, followed 90 min later by a 25-mg i.v. dose of diphenhydramine. Thirty minutes later, subjects were administered a 30-min i.v. infusion of 5 mg temsirolimus. Subjects remained in the clinical unit until the 72-h blood draw (study day 18) and returned for all subsequent blood draws. They continued to take 400 mg ketoconazole orally each day through study day 21. Subjects fasted for 10 h before receiving temsirolimus and for 4 h afterward.

Blood samples were collected predose, immediately before the end of the infusion (0.5 h), and at 1, 3, 8, 24, 48, 72, 96, 120, 144, and 168 h after administration of temsirolimus on study days 1 and 15. Samples were collected in evacuated tubes containing ethylenediaminetetraacetic acid (EDTA) and gently mixed, then stored upright at −70°C until shipment. Samples were shipped on ice to Taylor Technology (Princeton, NJ, USA) for measurement of temsirolimus and sirolimus concentrations.

### Bioanalytic assays

The concentrations of temsirolimus and sirolimus in blood were measured simultaneously using validated liquid chromatography/tandem mass spectrometry combination procedures with deuterated internal standard (Punt *et al*, 2003). Analytes and internal standard were extracted from a 1-ml sample of EDTA-treated whole blood by liquid–liquid extraction with 1-chlorobutane and separated with a YMC PDS AQ HPLC column (Waters, Milford, MA, USA). Analytes were detected by tandem mass spectrometry using atmospheric pressure chemical ionisation. The concentration of analytes was determined with calibration standards using linear least squares regression.

The bioanalytic method for temsirolimus was validated through the quantitation range of 0.25–100 ng ml^−1^ and exhibited interday and intraday variabilities, expressed as CV of <5% and biases of <9.4% (Punt *et al*, 2003). The bioanalytic method for sirolimus was validated through the quantitation range of 0.1–100 ng ml^−1^ and exhibited interday and intraday variabilities of <12.7% and biases of <11.3% (Punt *et al*, 2003). No interferences were observed in blank blood or in blood spiked with internal standard. Temsirolimus and sirolimus are stable in whole blood when stored at −70°C (Punt *et al*, 2003).

### Pharmacokinetics

Pharmacokinetic parameters for temsirolimus were analysed for each subject for each study period using non-compartmental methods (Jusko, 1992). Scheduled times were used for all calculations. The *C*_max_ and the time to reach *C*_max_ (*T*_max_) were determined visually from the concentration–time curves. The terminal elimination rate constant, *λ_z_*, was estimated by log-linear regression of the terminal portion of the whole blood concentration–time curves. The terminal elimination half-life (*T*_1/2_) was calculated as 0.693/*λ_z_*. The area under the concentration–time curve (AUC_*T*_) was calculated using the trapezoidal rule for the ascending portion of the curve, and the log-trapezoidal rule for the descending portion of the curve. Total AUC was estimated using the following equation: AUC=AUC_*T*_+*C*_*T*_/*λ_z_*, wherein *C*_*T*_ is the last observed blood concentration at time *T*. The AUC_ratio_ is the ratio of sirolimus AUC to temsirolimus AUC (AUC_sirolimus_/AUC_temsirolimus_). Clearance (CL) is the volume of plasma cleared of the drug per unit time (dose/AUC). Apparent clearance was corrected for the fraction of the dose that was metabolised (CL/*f*_m_). The apparent volume of distribution during the terminal phase (*V*_z_) was calculated by (CL/*f*_m_)/*λ_z_*. The calculation of the steady-state volume of distribution (*V*_dss_) incorporated dissociation and association rates of protein and tissue binding. *V*_dss_ was defined as (dose/AUC)MRT_u_, where MRT_u_ is the mean residence time of the unbound drug in the plasma (Berezhkovskiy, 2004).

### Statistics

The blood concentrations and PK parameters for temsirolimus and sirolimus from both treatment periods were compared using a one-factor analysis of variance (ANOVA) with treatment as a fixed effect and subject as a random effect, using the WinNonlin Enterprise application version 4.1 Linear Mixed Effects Wizard (Pharsight Corp., Mountain View, CA, USA). The geometric mean relative bioavailability of *C*_max_, AUC, and AUC_*T*_ and the 90% confidence limits were calculated using ANOVA to determine the magnitude of the effect of ketoconazole. Vital signs, ECG intervals, and laboratory parameters were screened for prespecified, potentially clinically important criteria.

## RESULTS

### Study population

Seventeen subjects were enrolled in the study, and all but one were men and most were white subjects (Table 1). During the study, 17 subjects received temsirolimus during period 1, whereas 14 subjects received temsirolimus with ketoconazole during period 2. Five subjects did not complete the study. During period 1, one subject received only a partial dose of temsirolimus and withdrew from the study after experiencing a local reaction to the i.v. infusion, for which an ice pack was applied and the infiltration was considered resolved. One other patient withdrew consent. During period 2, one patient failed to return and two other patients withdrew consent following period 2 treatment. Available data from 16 subjects were included in the PK analyses, and all 17 subjects were included in the safety analyses.

### Pharmacokinetics

The temsirolimus PK profile was evaluable for 16 subjects in period 1 and for 14 subjects in period 2. Exposures to temsirolimus and sirolimus were associated with a modest degree of intersubject variability, with a CV for *C*_max_ and AUC of <16% for temsirolimus and <33% for sirolimus.

**Figure 1:**
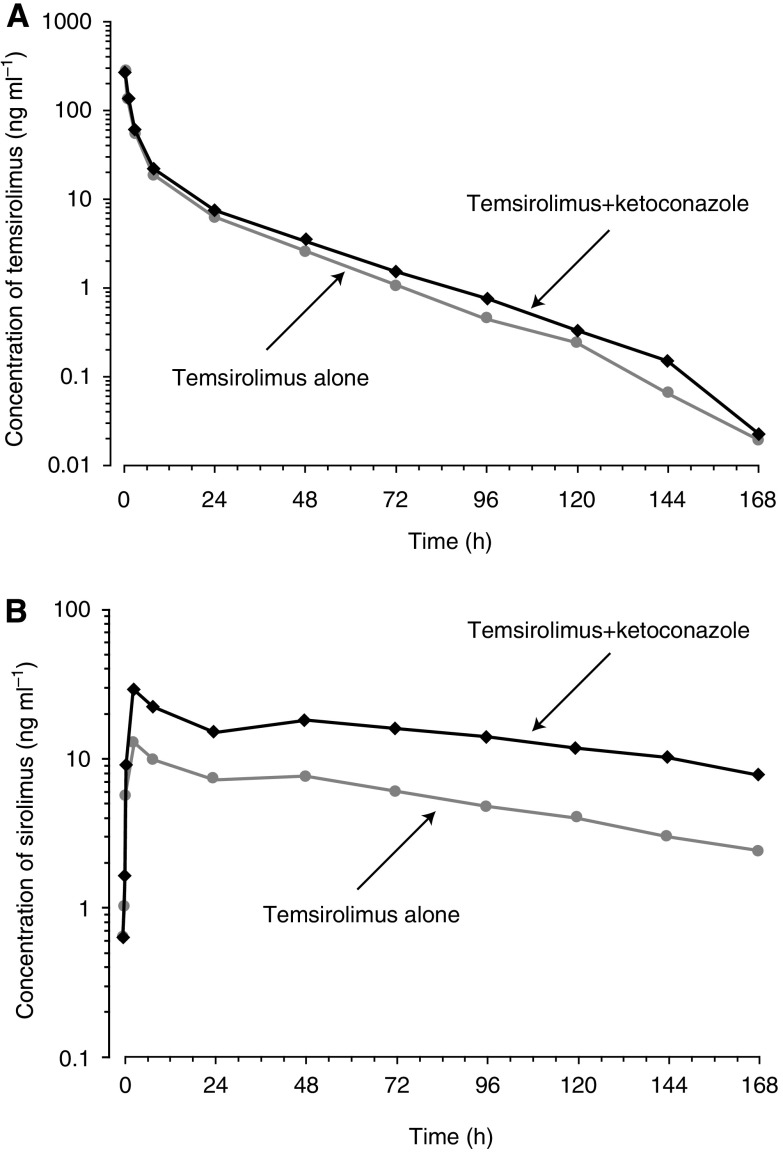
Mean concentration *vs* time profile of temsirolimus (**A**) and sirolimus (**B**) in whole blood following a single 5-mg i.v. dose of temsirolimus alone or with multiple doses of ketoconazole.

Mean concentration–time curves for temsirolimus in blood were nearly comparable following treatment with ketoconazole in combination with temsirolimus compared with temsirolimus alone (Figure 1A). In contrast, the mean concentration of sirolimus over time was greater in subjects receiving ketoconazole in combination with temsirolimus than that in subjects receiving temsirolimus alone (Figure 1B). Time to maximal concentration consistently occurred at the end of infusion (0.5 h) for temsirolimus and at 3 h for sirolimus, irrespective of treatment.

No substantial differences in mean *C*_max_ and AUC for temsirolimus were observed in subjects receiving temsirolimus alone or in combination with ketoconazole (Table 2). Mean volume of distribution of temsirolimus at steady state was 87 and 92  l without or with ketoconazole coadministration, respectively. Mean clearance of temsirolimus decreased from 5.72 to 5.14 l h^−1^ (approximately 10%), but this decrease was not manifested as changes in mean *C*_max_ and AUC.

In contrast, for sirolimus, the change in mean apparent metabolic clearance (CL/*f*_m_) was more substantial, decreasing 3.2-fold with ketoconazole treatment and resulting in an increase in mean half-life from 75 to 113 h. Carryover of sirolimus from treatment period 1 to 2 was minimal, contributing 1.9% to the mean total AUC calculated for this treatment period (range 1.1–3.7%). Ketoconazole increased sirolimus mean *C*_max_ by 2.2-fold and AUC by 3.2-fold compared with temsirolimus alone.

**Figure 2:**
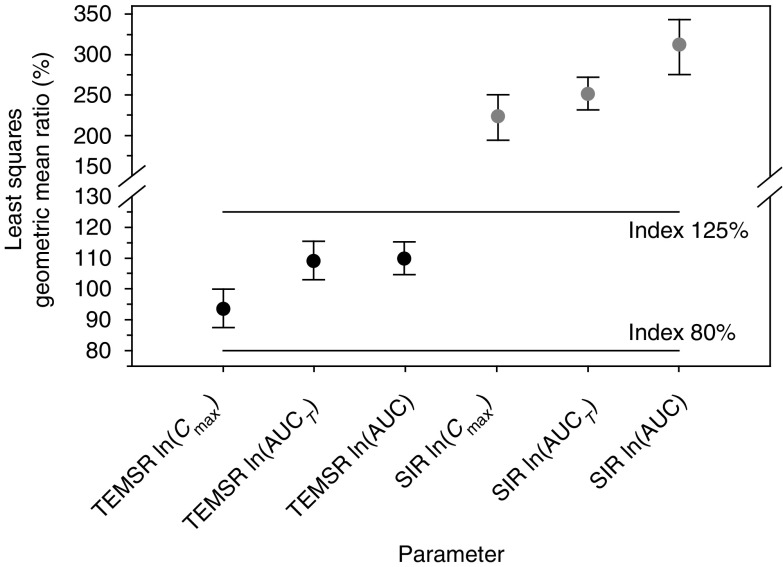
Least squares geometric mean ratios of temsirolimus (TEMSR) and sirolimus (SIR) *C*_max_, AUC_*T*_, and AUC following a single 5-mg i.v. dose of temsirolimus alone or with multiple doses of ketoconazole, in healthy subjects.

For temsirolimus, the least squares geometric mean ratios (90% confidence intervals (CIs)) relative to the reference of a single dose of 5-mg i.v. temsirolimus alone for *C*_max_, AUC_*T*_, and AUC were 94% (88%, 100%), 109% (103%, 116%), and 110% (105%, 115%), respectively. For sirolimus, the least squares geometric mean ratios (90% CIs) for *C*_max_, AUC_*T*_, and AUC were 224% (198%, 253%), 252% (232%, 272%), and 310% (277%, 345%), respectively (Figure 2).

### Safety

Adverse events, defined as those occurring or worsening after receiving study medications, occurred in 71 and 57% of subjects receiving temsirolimus and the combination of temsirolimus and ketoconazole, respectively (Table 3). The most frequently reported adverse events were headache, neutropaenia, pain, and insomnia. No clinically important trends in laboratory results, vital signs, or ECG intervals occurred between treatment groups. In fact, toxicity profiles were comparable between the temsirolimus plus ketoconazole and temsirolimus alone arms. No deaths or serious adverse events were reported.

## DISCUSSION

Temsirolimus is a novel targeted therapy for the treatment of patients with RCC and is currently in clinical studies for treatment of various haematologic and solid malignancies. Patients with advanced cancers often receive concomitant therapies to manage disease symptoms and the side effects associated with anticancer agents. It is therefore important to characterise the potential for PK drug interactions in patients receiving i.v. temsirolimus and to provide guidance on dose modifications, when appropriate.

We report that coadministration of ketoconazole, a strong CYP3A4 inhibitor, with 5 mg i.v. temsirolimus in healthy subjects had no effect on temsirolimus *C*_max_ or AUC. The 90% CIs for the least squares geometric mean ratios of *C*_max_, AUC_*T*_, and AUC for temsirolimus were within the 80–125% range, indicating no clinically significant PK drug interaction. However, for the metabolite sirolimus, 90% CIs for the least squares geometric mean ratios of *C*_max_, AUC_*T*_, and AUC were greater than 200%, indicating a clinically significant PK drug interaction. These increases in sirolimus *C*_max_ and AUC were accompanied by a 69% reduction in sirolimus apparent clearance.

The clinical relevance of increased exposure to sirolimus PK for patients with cancer receiving i.v. temsirolimus pertains to the potential for increased incidence or severity of toxicities rather than efficacy concerns. With 5 mg i.v. temsirolimus, ketoconazole increased the sirolimus/temsirolimus AUC_ratio_ 2.9-fold (from 1.35 to 3.94). Despite greater exposure to sirolimus, increases in toxicity were not evident in these healthy subjects. The most common adverse events were headache, neutropaenia, pain, and insomnia. Notwithstanding the lower 5 mg dose administered in this study, the types of adverse events observed were not dissimilar to those seen in studies of temsirolimus in patients with cancer, although a greater percentage of cancer patients had thrombocytopaenia, rash, and mucositis (Atkins *et al*, 2004; Hidalgo *et al*, 2006) than did the healthy subjects in this study. Previous studies have suggested that the incidence and severity of some adverse events are proportional to the exposure to temsirolimus (Boni *et al*, 2005; Chang *et al*, 2005). In a population PK safety analysis of patients receiving 25, 75, or 250 mg of temsirolimus weekly, clinically interesting associations between AUC_sum_ and adverse event severity were observed for thrombocytopaenia (*P*=0.007), pruritus (*P*=0.011), and hypertriglyceridaemia (*P*=0.040) (Boni *et al*, 2005). Based on both logistic regression analyses, where predicted drug concentrations can estimate the probability of adverse events, and the tolerability of temsirolimus over a wide dose range, the likelihood of severe toxicity occurring from this type of drug interaction is thought to be low with the 25-mg dose. However, if strong CYP3A4 inhibitors need to be used, a temsirolimus dose reduction to 12.5 mg should be considered.

Sirolimus appears to be more sensitive than temsirolimus to both strong CYP3A4 inhibitors and strong CYP3A4-inducing agents. Rifampin, a potent inducer of CYP3A4, had no effect on temsirolimus AUC but reduced sirolimus AUC by 56% in healthy adults (Boni *et al*, 2007). A study in cancer patients showed 36 and 67% reductions in *C*_max_ for temsirolimus and sirolimus, respectively, with concomitant enzyme-inducing anticonvulsant agents (such as carbamazepine, phenytoin, and clonazepam), as well as a 45% reduction in sirolimus AUC (Boni *et al*, 2007).

This is the first report of the effects of concomitant CYP3A4 inhibitors on the PK of temsirolimus. However, the effects of CYP3A4 inhibitors, including various macrolide antibiotics and azole antifungal medications, on the metabolism of oral sirolimus (rapamycin; Rapamune^®^, Wyeth Pharmaceuticals) have been previously described by Zimmerman (2004). Coadministration of ketoconazole with i.v. temsirolimus leads to increased blood levels of the sirolimus metabolite (3.1-fold); however, the magnitude of this increase was more profound with oral sirolimus (almost 10-fold) (Zimmerman, 2004). It is likely that ketoconazole-mediated inhibition of CYP3A4 metabolism in the gastrointestinal epithelium plays a significant role in enhancing first-pass absorption following oral administration of CYP3A4 substrates like sirolimus. Intravenous administration, as is employed with temsirolimus, largely avoids first-pass presentation of drug to the gut so that this mechanism has less of a bearing on drug interaction potential.

Temsirolimus and sirolimus are both substrates of CYP3A4, but neither is an inhibitor or an inducer of CYP3A4. Thus, the effect of i.v. temsirolimus administration on the metabolism of other CYP3A4 substrates should be minimal or negligible. These include HMG-CoA reductase inhibitors and glucose-lowering agents (commonly used to treat hyperlipidaemia and hyperglycaemia, respectively, that may be related to temsirolimus), benzodiazepines (commonly used in cancer patients for anxiety), and calcium channel blockers (commonly used antihypertensives). Although antifungal agents that are strong inhibitors of CYP3A4 (e.g., ketoconazole and itraconazole) should be avoided in patients receiving temsirolimus, moderate CYP3A4 inhibitors (e.g., fluconazole and voriconazole) may not have as much interaction potential. For example, in RCC patients treated with 25 mg i.v. temsirolimus, fluconazole (commonly used for treatment of chemotherapy-induced mucositis) may be a reasonable management approach for temsirolimus-related mucositis. Nevertheless, CYP3A4 inhibitors should not be given concomitantly with higher temsirolimus doses, such as those being investigated in clinical trials (e.g., 175 mg for mantle cell lymphoma).

In conclusion, coadministration of ketoconazole with i.v. temsirolimus had no effect on temsirolimus *C*_max_ or AUC; however, a strong PK interaction leading to increased exposure of the primary metabolite sirolimus (*C*_max_, 2.2-fold; AUC, 3.1-fold) was observed. Based on these results, it is recommended that concomitant use of strong CYP3A4 inhibitors should be avoided (e.g., ketoconazole, itraconazole, clarithromycin, atazanavir, indinavir, nefazodone, nelfinavir, ritonavir, saquinavir, telithromycin, and voriconazole) in cancer patients receiving 25 mg i.v. temsirolimus once weekly. Grapefruit juice may also increase plasma concentration of sirolimus. If a concomitant strong CYP3A4 inhibitor is necessary, a temsirolimus dose reduction to 12.5 mg weekly should be considered. If the strong CYP3A4 inhibitor is discontinued, a washout period of approximately 1 week should be allowed before the temsirolimus dose is adjusted back to the dose used before initiation of the strong CYP3A4 inhibitor.

## Figures and Tables

**Table 1:** Subject demographics and characteristics

**Characteristic**	**Total (*n*=17)**
Mean age (years)	33.7±8.9
*Gender (n (*%*))*	
Women	1 (5.9)
Men	16 (94.1)
Height (cm)	175.9±5.9
Weight (kg)	79.8±10.8
Body mass index (kg m^−2^)	25.8±3.1
*Ethnicity (n (*%*))*	
Black or African American	6 (35.3)
Other: multiracial	1 (5.9)
White	10 (58.8)

**Table 2:** Mean pharmacokinetic parameters of temsirolimus and sirolimus in whole blood

	**Temsirolimus, mean (CV)**		**Sirolimus, mean (CV)**	
**Pharmacokinetic parameter**	**Temsirolimus (5 mg)**	**Temsirolimus (5 mg)+ ketoconazole (400 mg)**	**Temsirolimus (5 mg)**	**Temsirolimus (5 mg)+ ketoconazole (400 mg)**
*C*_max_ (ng ml^−1^)	278.3 (5.8%)	262.6 (15.5%)	13.3 (27.4%)	29.0 (13.6%)
*T*_max_ (h)	0.5 (0)	0.5 (0)	3.3 (37.7%)	3.0 (0)
*T*_1/2_ (h)	22.3 (19.6%)	23.7 (17.0%)	74.8 (10.8%)	112.8 (18.1%)
AUC_*T*_ (h ng ml^−1^)	873.4 (11.9%)	969.1 (15.0%)	918.3 (28.3%)	2410.7 (22.0%)
AUC (h ng ml^−1^)	885.5 (11.7%)	991.6 (14.5%)	1204.7 (30.2%)	3888.9 (29.2%)
CL^2^ (l h^−1^)	5.7 (11.0%)	5.1 (13.8%)	4.5 (28.6%)	1.4 (32.7%)
*V*_z_^3^ (l)	182.0 (17.1%)	173.6 (16.4%)	479.7 (27.5%)	220.4 (24.5%)
*V*_ss_^4^ (l)	86.8 (14.6%)	91.5 (17.5%)	493.6 (25.4%)	227.1 (22.0%)
AUC_ratio_			1.35 (23.9%)	3.94 (28.0%)
AUC_sum_ (h ng ml^−1^)			2090 (21.0%)	4881 (25.0%)

AUC=area under the concentration curve; AUC_sum_=sum of temsirolimus plus sirolimus AUC; AUC_*T*_=area under the concentration–time curve; CL=clearance; *C*_max_=maximum concentration; *T*_1/2_=half-life; *T*_max_=time to reach *C*_max_; *V*_z_=apparent volume of distribution during the terminal phase; *V*_ss_=steady-state volume of distribution.

2 For sirolimus=CL/*f*_m_ (l h^−1^).

3 For sirolimus=*V*_z_/*f*_m_ (l).

4 For sirolimus=*V*_ss_/*f*_m_ (l).

**Table 3:** Treatment-emergent adverse events

**Body system adverse event, *n* (%)**	**Temsirolimus (5 mg) (*n*=17)**	**Temsirolimus (5 mg)+ketoconazole (400 mg) (*n*=14)**
Any adverse event	12 (71)	8 (57)
		
*Body as a whole*		
Back pain	1 (5.9)	0
Chills	0	1 (7.1)
Headache	4 (23.5)	2 (21.4)
Neck pain	1 (5.9)	0
Pain	2 (11.8)	0
		
*Cardiovascular system*		
Syncope	1 (5.9)	0
Tachycardia sinus	1 (5.9)	0
Vascular headache	0	1 (7.1)
		
*Digestive system*		
Aphthous stomatitis	0	1 (7.1)
Dry mouth	1 (5.9)	0
Dyspepsia	0	1 (7.1)
Mouth pain	1 (5.9)	0
Nausea	0	1 (7.1)
		
*Haemic and lymphatic system*		
Granulocytosis	1 (5.9)	0
Neutropaenia	3 (17.6)	1 (7.1)
		
*Nervous system*		
Hostility	0	1 (7.1)
Insomnia	1 (5.9)	0
Nervousness	0	1 (7.1)
Tremor	0	1 (7.1)
Twitching	0	1 (7.1)
		
*Respiratory system*		
Epitaxis	0	1 (7.1)
Rhinitis	1 (5.9)	0
		
*Skin and appendages*		
Herpes simplex	1 (5.9)	0
Rash	1 (5.9)	0
		
*Special senses*		
Visual field defect	0	1 (7.1)
		
*Adverse event associated with miscellaneous factors*		
Local reaction to procedure	1 (5.9)	0
